# Deciphering composition and function of the root microbiome of a legume plant

**DOI:** 10.1186/s40168-016-0220-z

**Published:** 2017-01-17

**Authors:** Kyle Hartman, Marcel GA van der Heijden, Valexia Roussely-Provent, Jean-Claude Walser, Klaus Schlaeppi

**Affiliations:** 1 Plant-Soil Interactions, Agroscope, Institute for Sustainability Sciences, Reckenholzstrasse 191, CH-8046 Zürich, Switzerland; 2 Department for Evolutionary Biology and Environmental Studies, University of Zürich, Zürich, Switzerland; 3 Plant-Microbe Interactions, Institute of Environmental Biology, Faculty of Science, Utrecht University, Utrecht, The Netherlands; 4 ISARA-Lyon, Lyon, France; 5 Genetic Diversity Centre, ETH Zürich, Zürich, Switzerland

**Keywords:** Clover, Root, Microbiome, 16S rRNA sequencing, Microcosm

## Abstract

**Background:**

Diverse assemblages of microbes colonize plant roots and collectively function as a microbiome. Earlier work has characterized the root microbiomes of numerous plant species, but little information is available for legumes despite their key role in numerous ecosystems including agricultural systems. Legumes form a root nodule symbiosis with nitrogen-fixing *Rhizobia* bacteria and thereby account for large, natural nitrogen inputs into soils. Here, we describe the root bacteria microbiome of the legume *Trifolium pratense* combining culture-dependent and independent methods. For a functional understanding of individual microbiome members and their impact on plant growth, we began to inoculate root microbiome members alone or in combination to *Trifolium* roots.

**Results:**

At a whole-root scale, *Rhizobia* bacteria accounted for ~70% of the root microbiome. Other enriched members included bacteria from the genera *Pantoea*, *Sphingomonas*, *Novosphingobium*, and *Pelomonas*. We built a reference stock of 200 bacteria isolates, and we found that they corresponded to ~20% of the abundant root microbiome members. We developed a microcosm system to conduct simplified microbiota inoculation experiments with plants. We observed that while an abundant root microbiome member reduced plant growth when inoculated alone, this negative effect was alleviated if this *Flavobacterium* was co-inoculated with other root microbiome members.

**Conclusions:**

The *Trifolium* root microbiome was dominated by nutrient-providing *Rhizobia* bacteria and enriched for bacteria from genera that may provide disease protection. First microbiota inoculation experiments indicated that individual community members can have plant growth compromising activities without being apparently pathogenic, and a more diverse root community can alleviate plant growth compromising activities of its individual members. A trait-based characterization of the reference stock bacteria will permit future microbiota manipulation experiments to decipher overall microbiome functioning and elucidate the biological mechanisms and interactions driving the observed effects. The presented reductionist experimental approach offers countless opportunities for future systematic and functional examinations of the plant root microbiome.

**Electronic supplementary material:**

The online version of this article (doi:10.1186/s40168-016-0220-z) contains supplementary material, which is available to authorized users.

## Background

Plant roots in soil are in contact with the most microbially diverse biome on the planet, with estimates of bacteria diversity as high as 38,000 taxa per gram of soil [1]. The root bacteria microbiome typically consists of *Proteobacteria*, *Actinobacteria*, and *Bacteroidetes* [2]. Recent studies have highlighted the root bacteria microbiome of several plant species, including *Arabidopsis* [3, 4] and a number of crop species, like barley [5], maize [6], sugarcane [7], and rice [8]. However, the microbiome of nitrogen-fixing plants, in particular legumes such as red clover, has received little attention in microbiome studies.

*Trifolium pratense* (red clover, hereafter: Trifolium) is an important forage legume and grown on approximately four million hectares worldwide [9]. Because of its beneficial symbiosis with N-fixing rhizobia, Trifolium is cultivated in grass/clover mixtures or as a cover crop in crop rotations [10]. While the species’ genetic diversity has been characterized using morphological traits [11], DNA marker polymorphism [12], and genome analyses [13], its root microbiome has not been investigated using high-throughput sequencing tools. Furthermore, Trifolium’s association with rhizobia suggests its microbiome may differ from non-legumes in that rhizobia are expected to be highly abundant [14].

The N-provision by rhizobia represents a well-established service to their host. Similarly, other microbiome members were found to assist their host plant in nutrient uptake, protection from pathogens, or modulating immunity responses [15, 16]. However, how microbial functions affect plants if a service-providing member is in a diverse community, and how entire microbial communities affect their host, remains poorly understood [16]. One limitation of ribosomal RNA-based root microbiota characterizations is that such approaches only provide indirect information, based upon taxonomic classification, about the function(s) of its members. One suggested approach for the functional examination of the root microbiome relies on isolating root microbes to build microbe collections [17]. The availability of bacterial isolates offers the opportunity for genome sequencing to obtain insights into their potential functions, but more importantly, the activity of these strains can be empirically tested in host-microbiota interaction experiments.

Microbe collections have been assembled [18–22] despite that the recalcitrance to cultivation of many bacteria taxa—with estimates that more than 99% of soil bacteria cannot be cultured [23]—was often seen as a limitation. This recalcitrance does not necessarily apply to bacteria of the root microbiome as evidenced by an earlier study of Chelius and Triplett [24], who reported a phylogenetic overlap of 48% between their bacteria isolate collection and a 16S ribosomal RNA (rRNA) clone library from maize roots. More recently, Bai et al. [21] reported a collection of nearly 6000 root-derived bacteria isolates and a remarkable 54–65% isolation rate compared to the abundant (>0.1% relative abundance) operational taxonomic units (OTUs) in *Arabidopsis thaliana* roots. However, it required considerable effort including large-scale isolation using serial dilutions (seven different bacteria isolation media were used!) and subsequent high-throughput taxonomy identification.

Experimental manipulation of the microbiome and assays with plants require contained systems in which host-microbiota interaction experiments can be conducted without outside microbial contamination. Recently, microcosm systems have been used in combination with bacteria reference stocks to examine the dynamic process of root microbiome assembly from a defined input community under microcosm conditions [21, 22]. In these experiments, stable and reproducible community assembly was observed. However, these experiments were not designed to clarify how root communities compare to plants grown in artificial substrate in microcosms or in natural soil conditions.

**Fig. 1:**
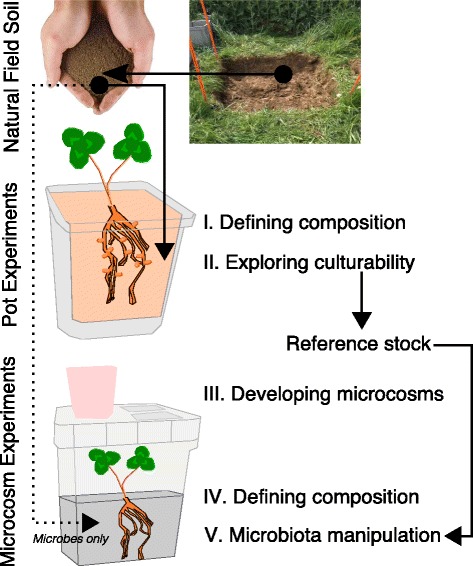
Characterization of the root microbiome. We collected a natural field soil and used it in a series of Trifolium growth experiments. (*I*) We investigated the composition of the root bacteria microbiome using 16S rRNA sequencing of root samples. (*II*) We utilized the same root material for an isolation effort to explore the culturable fraction of root bacteria microbiome and assembled a reference stock of bacteria isolates. (*III*) We subsequently developed a microcosm system to explore plant-microbiota interactions and (*IV*) investigated the composition of the Trifolium root microbiome in the system by inoculating microbiota extracted from the field soil. (*V*) We conducted microbiota manipulation experiments in which we inoculated culturable, abundant members of the root microbiome and scored their effects on plant growth

Here, we addressed some of the aforementioned research gaps and report a detailed characterization of the Trifolium root bacteria microbiome. We sampled the whole-root system including nodules, removed the rhizosphere and investigated the entire root bacterial communities consisting of rhizoplane and endosphere habitats. We utilized a multi-step approach to investigate the composition and culturable fraction of its root microbiome (Fig. 1). We also move towards a functional understanding of specific members of the Trifolium root microbiome and developed a microcosm system (Additional file 1: Figure S1a–d) in which we conducted multi-strain inoculation experiments with Trifolium germinated from surface-sterilized seeds and investigated the inoculation-induced effects on plant growth.

## Results

### Composition of the Trifolium root microbiome

The 16S amplicon sequencing of 24 Trifolium root samples and 15 soil samples from climate chamber and natural site growth experiments (Fig. 1, Table 1, Additional file 1: Figure S2,) yielded 9,923,925 high-quality, non-chimeric sequences across all samples, with a median of 153,072 (range 21,731–981,922) sequences per sample (Additional file 2). We rarefied the dataset to an even sequencing depth of 20,000 sequences and identified 3495 bacteria OTUs and one archaea OTU.

**Table 1:** Overview of the number of replicate samples by sample type, experiment, and experimental replicate, or plot

	Experimental soil	Natural site^a^			Climate chamber					
Sample	–	Plot 1	Plot 2	Plot 3	Ex^b^ 1	Ex 2^c^	Ex 3	Ex 4	Ex 5^c^	Microcosms
Root	–	3	3	3	3	3	3	3	3	8^d^/12^e^
Soil	3	3	3	3	3	–	–	–	–	3
Inoculum	–	–	–	–	–	–	–	–	–	4^f^/3^g^

^a^Bacteria isolates from natural site plants were cultured from plants collected from within and outside the experimental plots

^b^Experiment

^c^Bacteria isolates from climate chamber plants were cultured from these experiments, plus one non-sequenced growth experiment

^d^Total number of samples collected from the soil extract experiment. One root sample was collected from each replicate microcosm

^e^Total number of samples from the simplified community experiments. Four root samples were collected from each of the three experiments

^f^Independently prepared soil extract samples used as the experimental start inoculum. See Additional file 1 for details

^g^One inoculum sample for each microcosm experiment

**Fig. 2:**
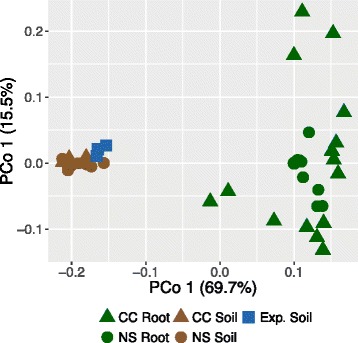
Sample type, growth conditions, and experiment explain much of the variation in soil and root bacteria communities. Unconstrained principal coordinates analysis (PCoA) of weighted UniFrac distances of root and soil samples from climate chamber (*CC Root*, *CC Soil*) and natural site growth experiments (*NS Root*, *NS Soil*), as well as the unplanted experimental field soil (*Exp. Soil*). See Additional file 1: Figure S4 for points colored by the replicate experiment

We confirmed in the Trifolium root microbiome the typical patterns that are often observed in microbial ecology. The soil microbiome is richer and phylogenetically more diverse than the root microbiome (Additional file 1: Figure S3; Table S1). We quantified the major components driving differences between samples (ß-diversity) using unconstrained principal coordinates analysis (PCoA) on weighted UniFrac distances and found a clear separation along axis 1 (explaining 69.7% of the overall variation) and confirmed the general pattern that soil and roots harbor distinct microbiomes (Fig. 2). Axis 2 explained 15.5% of the variation overall and separated mainly the root but not the soil samples, and we did not notice an obvious clustering whether the plants were grown in the same soil in a climate chamber or in the field, suggesting negligible effects of the *growth condition* on β-diversity*.* We detected a significant effect of *growth condition* on OTU richness only (Additional file 1: Figure S3; Table S1). However, experiment-to-experiment variation (especially climate chamber experiment 2) largely explained the variability between root samples (Additional file 1: Figure S4). Possible effects due to differences in climatic conditions were generally not detected and would have an effect size smaller than replicate experimental variation.

In the following, we break down the dissimilarities between soil and root samples to compositional patterns evident in the taxonomic profiles of the samples. Soil samples contained abundant *Proteobacteria*, *Actinobacteria*, and *Acidobacteria* accounting for a mean of 54.7, 24.7, and 6.9%, respectively (Additional file 1: Figure S5). The Trifolium root microbiome was dominated by *Proteobacteria* that accounted for a mean abundance of 90.7% across both experimental conditions (Additional file 1: Figure S5).

**Fig. 3:**
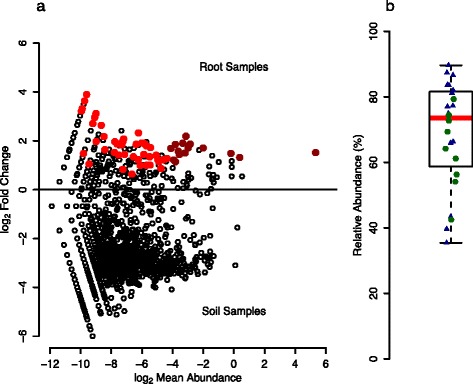
Abundant and root-specific OTUs of the Trifolium root microbiome. **a** The plot reports the mean relative abundance and the log_2_ fold change between root and soil samples of all OTUs present in the rarefied community (*open black circles*). *Filled red circles* indicate the 61 OTUs significantly enriched (*P* < 0.05, FDR corrected) in root samples. *Dark red circles* indicate the 15 OTUs present in the RootOTUs (see text). **b** Box plot (overplotted with individual data points) showing the median relative abundance of *OTU1* (*Rhizobium leguminosarum*) in sequenced climate chamber (*blue triangles*) and natural site (*green circles*) root samples

For the detailed characterization of the Trifolium root microbiome (Fig. 1, step I), we first identified the OTUs that were significantly higher in relative abundance in root compared to soil samples and discovered a total of 61 OTUs significantly enriched in root samples (Fig. 3), 15 of which were abundant with a mean relative abundance of at least 0.1% across all root samples. These 15 OTUs accounted for 74.5% of rarefied sequences, and we termed them “RootOTUs”—referring to the abundant and root-specific members of the Trifolium root microbiome. The RootOTUs consisted mostly of *Proteobacteria* (14 OTUs, Additional file 1: Table S2) and represented six different orders: *Rhizobiales* (6), *Sphingomonadales* (3), *Enterobacteriales* (2), *Burkholderiales* (1), *Caulobacterales* (1), and *Rhodospirillales* (1). The remaining non-*Proteobacteria* RootOTU belonged to the *Firmicutes* and was classified in the genus *Syntrophomonas.* We noted that one RootOTU (*OTU1*, matching *Rhizobium leguminosarum*) dominated the Trifolium root microbiome and explained the high prevalence of *Proteobacteria* (Additional file 1: Figure S5). *OTU1* ranged from 35.4 to 89.7% in samples from both growth conditions and accounted for a median of 73.5% of the root community (Fig. 3b). We confirmed that the high abundance of *OTU1* in the overall root community was due to the rhizobia bacteria present in root nodules (Additional file 1: Supplementary methods), and we noted a few non-*OTU1* sequences inside the nodules, suggesting additional within-nodule bacteria diversity (Additional file 1: Supplementary results, Figure S6).

In summary, root bacterial communities did not differ substantially whether the plants were grown under controlled or field conditions, thereby validating our approach using climate chamber experiments. The abundant and root-specific members of the Trifolium root microbiome consisted mainly of *Proteobacteria* and nodule-inhabiting rhizobia bacteria accounted for ~70% of the root microbiome.

### Isolated members of the Trifolium root microbiome

**Fig. 4:**
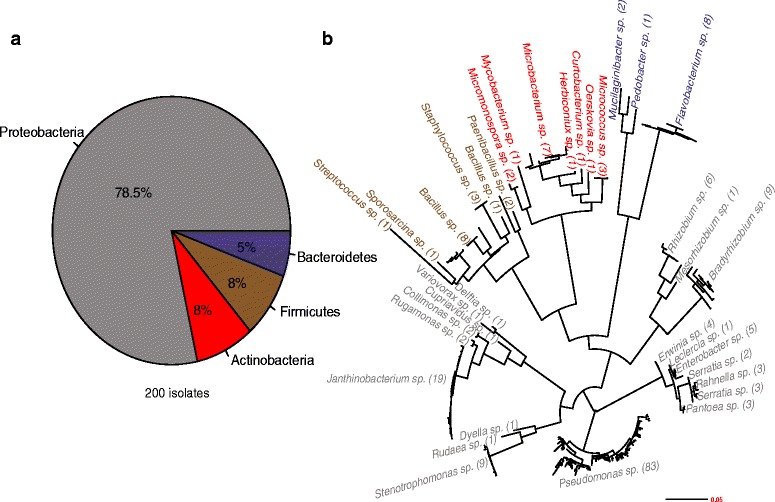
Taxonomic diversity of the Trifolium bacteria reference stock. **a** Taxonomic composition of the isolate collection at the Phylum level. **b** The phylogenetic diversity of the isolates at the genus level and the number of isolates assigned to each genus is indicated in parentheses. Isolates are labeled at the genus level and color-coded by phylum in **a**

We isolated bacteria from Trifolium roots of two climate chamber experiments and from plants grown at the natural site (Table 1) and characterized a total of 200 cultured bacteria (Fig. 1, step II). *Proteobacteria* dominated the culture collection, being represented by 78.5% isolates while *Actinobacteria*, *Firmicutes*, and *Bacteroidetes* accounted for 8, 8, and 5.5% of isolates, respectively (Fig. 4a). The isolates were assigned to 34 different genera (Fig. 4b). The 19 genera of the *Proteobacteria* (157 isolates) included abundant *Pseudomonas* (83 isolates), *Janthinobacterium* (19), and *Stenotrophomonas* (9). We found seven genera in the phylum *Actinobacteria* (16 isolates) with *Microbacterium* (7), *Micrococcus* (3), and *Micromonospora* (2) having more than one representative isolate. In the *Firmicutes* (16 isolates), we noted five different genera, with *Bacillus* (9), *Staphylococcus* (3), and *Paenibacillus* (2) being the most abundant. Finally, we found three genera in the *Bacteroidetes* (11 isolates): *Flavobacterium* (8), *Mucilaginibacter* (2), and *Pedobacter* (1).

**Fig. 5:**
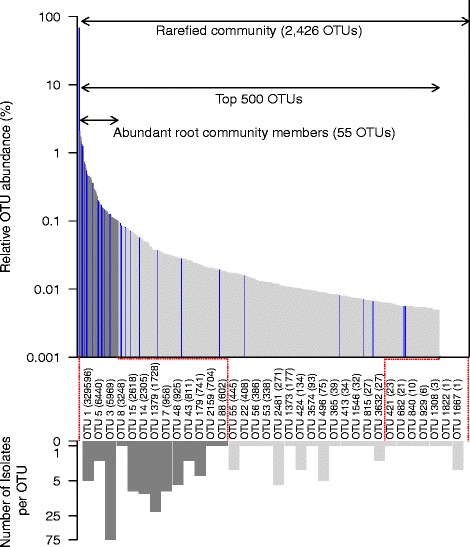
Mapping of reference stock bacteria to root microbiome OTUs. The *upper bar graph* represents the relative abundance of the 2426 OTUs in the root-associated bacteria community of Trifolium, with the 500 most abundant OTUs shown in *gray bars*. The *dark gray bars* indicate the 55 most abundant root OTUs (mean RA >0.1%). The *blue bars* indicate OTUs for which at least one isolate is present in the reference stock. The *lower, inverted bar graph* indicates the number of isolates in the reference stock mapping to an OTU in the community profile. *Bars* are shaded the same as in the upper graph to indicate the relative abundance of each OTU. *Bars* are labeled with the representative OTU name and its total number of sequences in the community profile in parentheses

We clustered the bacteria isolate sequences to the representative sequences of the OTUs of the Trifolium root community profiles at ≥97% sequence similarity (see Additional file 1: Supplementary methods) and determined whether a bacteria isolate constituted an abundant and root-enriched member of the Trifolium microbiome. Overall, out of the 200 bacteria isolates, 181 (90.5%) isolates clustered to 34 OTUs of the root community profile while for 19 (8.5%) isolates, we did not find a matching community member. All of the 34 isolated OTUs were present in the rarefied root community (2426 OTUs), corresponding to an isolation rate of 1.4% (Fig. 5). The isolation rate increased to 23.6% when comparing to the abundant community members: 55 abundant OTUs had a mean relative abundance of ≥0.1% across all root samples, and for 13 of these, we were able to culture bacteria strains. We identified 11 bacteria isolates for 2 of the 15 RootOTUs (Fig. 3; Additional file 1: Table S2). The cultured RootOTUs included the dominant *OTU1* (*R. leguminosarum*; 5 isolates), as well as *OTU48* (*Pantoea agglomerans*; 6 isolates).

We concluded that almost a quarter of the abundant root community members can be obtained in culture, and we achieved this with a manageable effort (200 strains) and straightforward microbiological techniques. By linking to the information of the root community profiles, we have characterized the bacteria strains of the reference stock with rank and relative abundance in the Trifolium root microbiome, and thereby the reference stock represents a toolbox for future microbiota manipulation experiments.

### Towards functional investigations of the Trifolium root microbiota

Finally, we developed microcosms (Fig. 1, step III) and evaluated their potential to conduct plant-microbiota interaction experiments. Recent microbiota inoculation experiments [21, 22] revealed that approximately half of the inoculated bacteria strains previously isolated from roots of soil-grown *Arabidopsis* either completely failed or failed to robustly colonize the roots of their host plant under microcosm conditions. We speculate that this could partly be due to the different physical and chemical conditions in the microcosms compared to soil and that these conditions are unfavorable for certain isolates. Therefore, we performed a soil extract experiment to pre-screen for possible microcosm-adapted bacteria strains. For this, we characterized the root microbiome of Trifolium that assembled after inoculation of a diverse soil microbiota extracted from the experimental field soil (Additional file 1: Figure S7a, b, Figure S8; Supplementary methods and results). We defined the root bacteria community (Fig. 1, step IV) and determined which bacteria isolates (from the reference stock, Fig. 5) corresponded to abundant OTUs on the roots under microcosm conditions (Additional file 1: Figure S8; Supplementary methods). See the Additional file 1: Supplementary results for a comparison between microcosm and soil-grown root communities (Additional file 1: Figure S9a, b; Supplementary methods and results).

We then conducted microcosm experiments in which we inoculated Trifolium in the microcosms with bacteria strains isolated from its root microbiome. The goal was not to screen strains or to test specific functions but instead to combine all our tools (reference stock, microcosms, community sequencing, and soil extract information) and validate the overall experimental approach for future microbiota inoculation experiments. We assembled a simplified community, choosing strains from the reference stock that corresponded to abundant OTUs on the roots under microcosm conditions *and* belonged to well-represented bacterial genera in the collection (Additional file 1: Figure S9; strains per OTU were randomly chosen): a *Flavobacterium* (*F*; *Bacteroidetes*, #8 isolates for this genus in the reference stock; KHB002), a *Pseudomonas* (*P; Proteobacteria*, #83; KHB004), and a *Janthinobacterium* (*J*; *Proteobacteria,* #19, KHB023; Table 2). We also included a *Microbacterium* (*M*; *Actinobacteria*, #7; strain KHB073) because this genus was well-represented in the reference stock (numerous isolates could indicate that these bacteria were abundant on roots; Fig. 4b) and because we wanted the inoculated community to broadly reflect the abundant bacterial phyla of plant root microbiomes (*Actinobacteria*, *Bacteroidetes*, and *Proteobacteria*; [2, 25]). We inoculated these bacteria alone or in combination to the autoclaved microcosms (Fig. 1, step V) at densities of 10^6^ cells mL^−1^ and planted surface-sterilized Trifolium seeds. We then monitored the community dynamics of the inoculated simplified community and scored effects of the bacteria inoculation on plant growth in three replicate experiments.

**Table 2:** Bacterial strains used in the microcosm experiments

Strain ID	Phylum	Genus	Species^a^	Abb.	OTU	Colonization^b^
Control	–	–	–	NBC	–	>1 × 10^2c^
KHB073	Actinobacteria	Microbacterium	*M. sp.* or *oxydans*	M	n.d.	7.80 × 10^6^
KHB002	Bacteroidetes	Flavobacterium	*F. succinicans*	F	OTU_7	3.51 × 10^6^
KHB004	Proteobacteria	Pseudomonas	*P. veronii* or *fluorescens*	P	OTU_3	5.48 × 10^7^
KHB023	Proteobacteria	Janthinobacterium	*J. lividum*	J	OTU_1379	2.95 × 10^7^

*Abb.* Abbreviation, *n.d.* not detected in the Trifolium root microbiome using high-throughput sequencing

^a^Taxonomy based on Greengenes 16S database [51]

^b^Mean bacterial cell number on roots after 25 days in the microcosms in experiment 3

^c^Highest order of magnitude at which observed OTUs were recorded

**Fig. 6:**
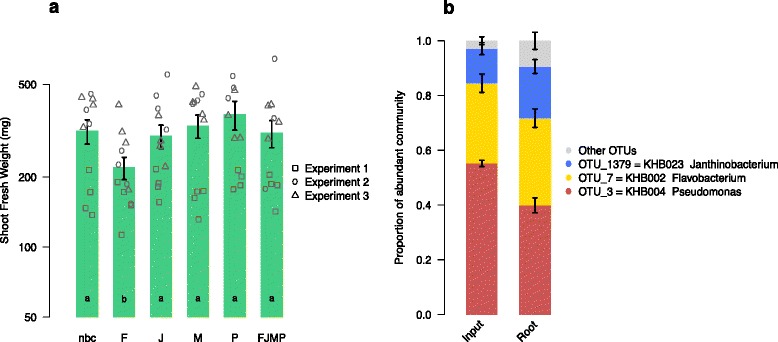
Functional analysis of a simplified Trifolium root microbiota in microcosms. **a** Trifolium growth in microcosms in the absence of inoculated bacteria (*nbc* no-bacteria control), with specific strains (*F* Flavobacterium KHB002, *J* Janthinobacterium KHB023, *M* Microbacterium strain KHB073, *P* Pseudomonas KHB004) or the simplified community (*FJMP*). The graph reports the mean shoot fresh weight (*n* = 12; ± s.e.m.) and the individual data points from the three independent experiments with four replicates each. The *letters* indicate statistical significance at *P* < 0.05 (Tukey’s HSD; analysis over the three experiments). Note, the Microbacterium (*M*, panel **a**) was not captured with the community quantification method. **b** Community composition of the simplified community (*FJMP*) at inoculation (input) and after 25 days on the roots. Sequences of other OTUs are indicated in *gray*

After 25 days, we harvested the experiments and counted ≥10^6^ bacterial colony forming units of the inoculated strains on the roots (Table 2). This confirmed that the chosen strains are also able to successfully colonize roots under the artificial growth conditions in the microcosms. We noted a lower biomass in one experiment compared to the two others, and this experiment-to-experiment variation indicated to us that numerous replicates are also needed when highly controlled conditions are used. With regard to the effects of individual bacteria inoculation on the plants, we found that the *Flavobacterium* negatively affected the growth of Trifolium, while the other bacteria did not have an effect on shoot biomass production (Fig. 6a). The combined application of the bacteria (*FJMP*) also did not have an apparent effect on biomass production but alleviated the negative impact of the *Flavobacterium* when grown alone. We measured the composition of the simplified community upon inoculation and after 25 days on the roots (Additional file 1: Supplementary methods for details). The *Microbacterium* could not be captured with the community quantification method, and we noted a small proportion of additional OTU sequences possibly representing sequencing errors or contamination, or in root samples, being derived from seed endophytes. Despite these limitations, the analysis revealed that the three other inoculated members retained similar proportions on the roots during 25 days of incubation as compared to when they were inoculated (Fig. 6b). This observation indicated that the alleviation of the negative impact of the *Flavobacterium* was not due to out-competition of this community member, but rather that its negative activities may have been “buffered” by the other bacteria in the simplified community.

## Discussion

### Root microbiome composition

Here, we have characterized the bacterial communities on roots of *T. pratense* with respect to their composition and reported first steps towards experimentally testing their functions. Trifolium harbors a diverse root microbiome that differs qualitatively and quantitatively from that of the surrounding bulk soil (Fig. 2), confirming studies with other plant species [3–5, 26]. We found that *OTU1*, matching *R. leguminosarum*, accounted for a median 73.5% of the root microbiome (Fig. 3b). We separately inspected root nodules and confirmed that Trifolium nodules were primarily inhabited by *R. leguminosarum* (Additional file 1: Figure S6) but also contained other bacteria taxa. This is in agreement with earlier work revealing within-nodule diversity in *Trifolium repens* and *Trifolium fragiferum*, which consisted of the dominant *R. leguminosarum* and the less-frequent rhizobia species *Bradyrhizobium japonicum*, *Sinorhizobium sp*., and *Mesorhizobium* [27, 28]. For the purpose of the microcosm experiments, we described the root microbiome of Trifolium at a whole-root scale, sampling the entire root system including nodules. For a broader description of legume microbiomes, future work investigating the variation in multiple soil types and comparisons with non-legume plants is needed. Additionally, an in-depth spatial assessment of legume root microbiomes would be insightful, e.g., by profiling the bacteria communities of root tissues with the nodules removed as well as inside the root nodules.

The large number of DNA sequences allowed us to thoroughly characterize the Trifolium root microbiome beyond the dominant rhizobia members. In addition to *Rhizobium*, Trifolium supports enriched OTUs from the genera *Pantoea*, *Sphingomonas*, *Novosphingobium*, and *Pelomonas*, among others, in its root microbiome (Additional file 1: Table S2). A review of relevant literature reveals that bacteria isolates of some of these genera have been found to be antagonistic to pathogens (Additional file 1: Table S2). This could possibly suggest a partitioning of complementary host services in the Trifolium root microbiome with “disease protection” and “nutrient provision” provided by the mentioned root-enriched genera and the nodule-inhabiting *Rhizobia*, respectively. However, because it is notoriously problematic to infer bacteria function from a taxonomy assignment [29], approaches other than 16S community sequencing are required for the functional understanding of the root microbiome. As a next step, such an indicative observation from cultivation independent microbiome analysis could be examined by testing reference stock bacteria belonging to these OTUs for their ability to suppress pathogens.

### Reference stocks and microcosms to study functions of the root microbiome

With the isolation of root microbiome members (Fig. 5), setting up an experimental microcosm system (Additional file 1: Figure S1a–d) and testing for microbiota effects on plant growth (Fig. 6a), we delineate a possible approach to advance the functional understanding of the root microbiome. We built our reference stock (Fig. 4b) using one bacteria isolation medium, and at a sampling depth of 200 bacteria strains, we captured close to a quarter of the abundant members of the Trifolium root microbiome. Therefore, we believe that our work presents an encouraging example especially for smaller laboratories with limited resources. For future work, additional isolation media and growth conditions would likely permit us to broaden the reference stock and contribute to a targeted cultivation of “missing” Trifolium root microbiome members.

### Experimentation with inoculated plants

We conducted multi-strain inoculation experiments with members of the Trifolium root microbiome to evaluate the suitability of microcosm growth system for plant-microbiota inoculation experiments. However, we first conducted the soil extract experiment (Additional file 1: Figure S7a, b, Figure S8; Supplementary methods and results) as a proof-of-concept to pre-screen microcosm-adapted bacteria strains. We subsequently tested four bacteria strains, three of which were culturable members of the abundant root community (Fig. 5) and were also abundant members of the root microbiome in the soil extract experiment (Additional file 1: Figure S8). We chose to include a *Microbacterium* isolate because of its abundance in our reference stock (seven isolates, Fig. 4b) and its classification in the *Actinobacteria*, a phylum shown to be abundant in plant root microbiomes [25]. We confirmed that these strains successfully colonized plant roots as suggested by the higher abundances on roots compared to their initial inoculated density to the microcosms (Table 2).

We could not capture the *Microbacterium* strain with the community quantification method (Fig. 6b), and similarly, none of the seven isolates from the reference stock clustered to any OTU in the entire dataset. A first possible explanation is that the *Microbacterium* is a rare but easily culturable microbiome member. Alternatively, the *Microbacterium* could be an abundant microbiome member, as indicated by the numerous isolates in the reference stock, but absent in the community profiles because of an observed mismatch in the priming site of the PCR primer 799F. A third possible explanation for the microcosms is that although the titer quantification revealed that the *Microbacterium* strain successfully colonized the plant roots in mono-associations, this strain was outcompeted in the simplified community by the other tested strains. Future experiments need to clarify these possibilities, but nevertheless, this is an example where cultivation and DNA-based approaches do not overlap, and a reminder that both methods have inherent limitations. While it is often discussed that PCR primers are biased towards certain bacterial taxa [30], the same is also true for isolation media, which have a specificity by favoring growth of certain bacterial groups [31].

We quantified the fresh weight of the shoot biomass in response to the bacteria in mono-associations or when the four bacteria were combined to a simplified community. We found that plants grew smaller when inoculated with the *Flavobacterium* strain in a mono-association, but that this negative plant growth response was alleviated when the *Flavobacterium* was inoculated in a community with the other strains (Fig. 6a). Since we measured that the *Flavobacterium* comprised roughly a third of the community (Fig. 6b), we excluded the possibility that the loss of the negative growth effect was due to the bacterium being outcompeted by the other inoculated strains. Instead, the growth compromising activities of the *Flavobacterium* were possibly counteracted by one or more of the co-inoculated isolates, or alternatively, it did not reach a sufficient cell density in the simplified community treatment.

The reference stock bacteria and microcosms present valuable resources for future microbiota manipulation experiments in which the contribution of the plant root microbiome to plant growth can be investigated. One next step would be to identify the functional traits, e.g., related to bio-control or plant growth promotion, of the reference stock bacteria using bioassays and/or genome sequencing. We expect that different strains that mapping to the same OTU would interact differently with the host plant, and thus the testing of the functional range among bacteria within an OTU presents another next step. In summary, there are countless opportunities for microcosm experiments. For example, the microbiota of Trifolium can be manipulated with regard to its taxonomic or trait composition or with regard to its diversity and tested for effects on plant growth. Furthermore, the interplay among community members or the dynamics of community assembly can be examined in more detail. Finally, microbiota induced effects on plant growth under stress conditions such as high salinity, reduced nutrient availability, or pathogens can be investigated.

## Conclusions

We have reported a multi-step approach (Fig. 1) combining cultivation-dependent and independent methods to describe and functionally examine the root microbiome of Trifolium. The need to experimentally manipulate a microbiota requires reference stocks of isolates, and we believe that reductionist plant-microbiota systems will permit a systematic examination of the root microbiome functions. Further studies employing targeted manipulations of the root microbiome can help in the development of new tools to increase the sustainability of other agricultural plant species [17] and investigate the relationship between microbiome diversity and plant performance [16].

## Methods

### Preparation of experimental soil, plant cultivation, and harvest

#### Experimental soil

All experiments of this study were conducted with a natural experimental soil collected from the area outside the experimental plots of the long-term Farming Systems and Tillage (FAST) experiment (47° 26′ 20″ N 8° 31′ 40″ E). The experimental soil is a loamy sand with the following physicochemical characteristics: pH 6.11; 16/31/51% clay/silt/sand; 19.37/1.25/4.88 mg/kg N/P/K (measured in 1:10 water extract by Eric Schweizer AG, Thun, Switzerland). In March 2013, we manually excavated three 1 m^2^ plots to a depth of 30 cm. The top layer of vegetation (5 cm) was removed, and the remaining bulk soil was collected, passed through a 2-mm sieve, homogenized and stored at 4 °C until use.

#### Plants

Seeds of *T. pratense* var. Milvus were surface-sterilized (10 min. in 70% ethanol, then 10 min. in 5% bleach and two washes with sterile H_2_O) and cultivated under controlled conditions (16 h/25 °C days, 8 h/16 °C nights; Additional file 1: Table S3) in climate chambers (Sanyo MLR-352H; Panasonic, Osaka, Japan) and natural conditions in a field experiment. For the climate chamber experiments, pots (8 × 8 × 8.5 cm) were filled with experimental soil, 15–20 sterilized seeds were sown in the center of each pot, and after 1 week of growth, the germinated seedlings were thinned until one plant per pot remained. The plants were watered two to three times per week with distilled H_2_O. We conducted five independent replicate climate chamber growth experiments (Additional file 1: Figure S2). We also conducted a field experiment in April 2013 using the three excavated plots from the soil collection effort (see above). A polycarbonate plastic ring (*∅* 30 cm, height 20 cm) was placed in the center of each plot and filled with the experimental soil (homogenized, sieved to 2 mm). The remaining area outside the plastic ring was filled with regular field soil. A few sterilized seeds were sown in each plot and covered with a thin layer of experimental soil (Additional file 1: Figure S2). During the growth period, the plots were weeded twice but otherwise exposed to natural conditions and not managed.

#### Harvest

The climate chamber plants were harvested after 9 weeks, and the field experiment was harvested once the plants reached the same growth stage as the plants in the climate chamber (14 weeks, Additional file 1: Figure S2). The entire soil volume inside the plastic ring with the aboveground plants was harvested and brought to the laboratory where the plants were processed. The roots were shaken to remove bulk soil and rinsed with distilled H_2_O to remove the rhizosphere (adhering soil particles), and we then sampled the 5-cm fragment of the root system corresponding to the soil depth between −1 and −6 cm using a scalpel in a Petri dish. The 5-cm root fragment presented the same sampling unit used for DNA extraction and for isolation of bacteria. Because our sampling method does not discriminate between microbes inhabiting the inner root tissue, root nodules, or the root surface, we refer to the profiled community as “root”-associated or simply “root” microbiome and do not differentiate between the different compartments. We also collected soil aliquots of the climate chamber and plots of the field experiment by sampling plant root-free bulk soil into 2-mL plastic tubes. The soil samples were flash-frozen in liquid nitrogen and stored at −20 °C until further processing.

### 16S rRNA community profiling

Detailed information regarding the sequencing approach is available in Additional file 1: Supplementary methods.

#### DNA extraction

Three 5-cm root fragments were combined into a 15-mL plastic tube making up one DNA sample, and we prepared three replicate DNA samples per experiment (nine root samples total). Similarly, for the field experiment, nine plants per plot were sampled and divided equally to make three replicate samples per plot. DNA was extracted using the FastDNA® SPIN Kit for Soil (MP Biomedicals, Solon, OH, USA) according to the manufacturer’s instructions (Additional file 1: Supplementary methods for further details).

#### PCR, library preparation, and sequencing

We used the primers 799F [24] and 1193R [32] flanking the variable regions V5–V7 of the 16S rRNA gene [33]. The 5′ end of the forward primer was amended with a unique 6-mer barcode selected from Faircloth and Glenn [34] (Additional file 2). See Additional file 1: Supplementary methods for details related to PCR and purification. Library preparation and sequencing were conducted at the Functional Genomics Centre Zurich (http://www.fgcz.ch) on the Illumina MiSeq Personal Sequencer (Illumina, San Diego, CA, USA).

#### Sequence processing

The raw reads were processed using an in-house-developed bioinformatics pipeline, which is available in Additional file 3. Briefly, the raw paired-end reads were quality filtered and trimmed at the 3′-end to 280 bp using PRINSEQ v0.20.4 [35] to improve the merging success and reduce error rate [36]. The trimmed paired-end reads were merged with FLASH v.1.2.9 [37]. Sequences from individual samples were de-multiplexed according to the forward barcode using Cutadapt v1.4.2 [38]. The merged 16S sequences were quality filtered with PRINSEQ and for OTU delineation truncated at a fixed length of 360 bp, sorted by abundance, de-replicated, and clustered to operational taxonomic units (OTU, ≥97% sequence similarity, singletons removed) with UPARSE v8.0.1623 [39]. Amplicons were chimera-screened against the GOLD database v.5 [40] and removed. Taxonomy assignment of the OTU representative sequences was performed using the SILVA 16S v119 database [41] with the RDP classifier as implemented in QIIME v1.8 [42].

### Statistical analysis of community profiles

All analyses were performed using R v3.1.2 [43] and different R packages. The R code and input files required to replicate all analyses and figures is available in Additional file 4, and the approach is outlined in Additional file 1: Supplementary methods. Briefly, the OTU and taxonomy tables were filtered to exclude OTUs classified as eukaryotes, chloroplasts, and mitochondria. The OTU table was rarefied to 20,000 sequences per sample (Additional file 1: Supplementary methods, Figure S10), and the abundance of each OTU was expressed as percentages of the total number of counts in a sample. All statistical analyses were performed on log_2_ + 1 transformed data. All *P* values were adjusted for multiple comparisons with the false discovery rate (FDR) correction using the Benjamini-Hochberg method [44]. We made use of the R packages *vegan* v2.3-5 [45], *picante* v1.6-2 [46], and the Bioconductor package *phyloseq* v1.14 [47].

### Bacteria reference stock

Detailed information regarding isolation, sequencing, and taxonomic assignment of bacteria isolates is available in Additional file 1: Supplementary methods.

We isolated root-associated bacteria from two climate chamber experiments and from Trifolium individuals collected from the field site by plating serial dilutions of a root slurry onto flour medium agar [48] plates amended with 10 μg mL^−1^ cycloheximide (to inhibit fungal growth; Sigma Aldrich, St. Louis, MO, USA). DNA extracted from single colony isolates was subjected to PCR using the primers 27F [49] and 1401R [50] and Sanger sequenced with 1401R as the sequencing primer by Microsynth AG (Balgach, Switzerland). These sequences were used for taxonomy assignment using the RDP classifier against the SILVA (v119) [41] database as implemented in QIIME [42]. Twenty-three isolates could not be assigned using SILVA and were further classified against the 16S ribosomal RNA database using NCBI BLAST. Additional file 5 gives the unique ID, source of isolation, taxonomy information, and 16S rRNA sequence and for each isolate.

### Microcosm experiments

Detailed information regarding the design of the microcosms and bacteria community experiments is available in Additional file 1: Supplementary methods.

We constructed experimental microcosms from Magenta GA-7 boxes (Sigma Aldrich, St. Louis, MO, USA) and filled them with 70 g of a calcined clay marketed as OilDri (Damolin GmbH, Oberhausen, Germany) (Additional file 1: Supplementary methods, Figure S1a, b). Microcosms containing the artificial soil substitute were covered with aluminum foil and sterilized by autoclaving (2 × 99 min at 121 °C). We pre-germinated surface-sterilized Trifolium seeds (see above) for 4 days under controlled conditions in a climate chamber (Additional file 1: Table S3) on square Petri dishes containing 0.5× Murashige and Skoog basal medium (Sigma Aldrich, St. Louis, MO, USA) supplemented with 1% sucrose. Seedlings with roots of ~1 cm length that were free of visible contaminations, but potentially containing endophytes, were used to conduct a microcosm experiment to assess the effect of four bacteria strains, inoculated individually and in combination, on plant growth (Additional file 1: Figure S1c, d). We determined the community profiles of the start inoculum of the combination treatment samples (three independent preparations) and the root samples using the 16S rRNA sequencing approach described above. The sequences of samples from all microcosm experiments were co-clustered with the sequences of the field- and climate chamber-grown Trifolium for community comparisons across experiments. We subsequently assessed the effect of the bacteria treatments on plant shoot biomass in the three replicate experiments using two-way analysis of variance (ANOVA). Significant differences between the different treatments were assessed with Tukey’s honest significant differences (HSD) test and were considered significant at *P* < 0.05.

## Additional files

Additional file 1: Supplementary methods. Expanded description of all experimental methods. Supplementary results. Results of the clone library analysis and soil extract microcosm experiment. Supplementary discussion. Discussion of root microbiome assembly in microcosms. **Figure S1.** Photos documenting the setup and planting of the microcosm experiments. **Figure S2.** Time-course photos of climate chamber and natural site Trifolium growth experiments. **Figure S3.** Rarefaction curves and α-diversity of root and soil samples in both growth conditions. **Figure S4.** PCoA plot colored individually by replicate Trifolium growth experiment. See Fig. 2 in the main text. **Figure S5.** Weighted UniFrac clustering of Trifolium root and soil samples linked to differences in phyla abundances. **Figure S6.** Clone library sequences clustering to OTUs from the root community profiles. **Figure S7.** Quantitative and qualitative comparisons of soil extract inoculum α-diversity to native field soil. **Figure S8.** Abundant root OTUs in the soil extract microcosm experiment. **Figure S9.** PCoA plot of inoculum, substrate, and root samples from the soil extract microcosm experiment clustered with native field soil and climate chamber root samples and a comparison between microcosm and climate chamber root communities. **Figure S10.** Box plot of sequencing depth across climate chamber and natural site root and soil samples. **Table S1.** ANOVA table of α-diversity analysis. **Table S2.** Taxonomy, OTU ID, and counts of Trifolium RootOTUs with potential genus function and literature references. **Table S3.** Temperature and light program used in the climate chamber growth and microcosm experiments. **Table S4.** PCR cycling conditions used in generating amplicons for MiSeq, isolate analysis, and the clone library. (DOCX 2200 kb)
Additional file 2: Sample name, experiment, barcode sequences, and sequence counts of the Trifolium root and soil samples and the simplified community and soil extract microcosm experiments. (XLSX 47 kb)
Additional file 3: Command line code and necessary input files needed to replicate bioinformatic analysis. (RAR 145 kb)
Additional file 4: R code and necessary input files needed to replicate all statistical analyses and reproduce R-generated figures. (RAR 2550 kb)
Additional file 5: Unique ID, taxonomy, isolation source, and FASTA sequence of the isolates in the bacteria reference stock. (XLSX 88 kb)

## Abbreviations

ANOVA: Analysis of variance
FAST: Farming Systems and Tillage
FDR: False discovery rate
HSD: Honest significant differences
OTU: Operational taxonomic unit
PCoA: Principal coordinates analysis
