# Supplementary material for: Deciphering composition and function of the root microbiome of a legume plant
Source: Microbiome. 2017 Jan 17;5:2. doi: 10.1186/s40168-016-0220-z (PMC5240445; doi:10.1186/s40168-016-0220-z)
Supplement: Additional file 1: — Supplementary methods. Expanded description of all experimental methods. Supplementary results. Results of the clone library analysis and soil extract microcosm experiment. Supplementary discussion. Discussion of root microbiome assembly in microcosms. Figure S1. Photos documenting the setup and planting of the microcosm experiments. Figure S2. Time-course photos of climate chamber and natural site Trifolium growth experiments. Figure S3. Rarefaction curves and α-diversity of root and soil samples in both growth conditions. Figure S4. PCoA plot colored individually by replicate Trifolium growth experiment. See Fig. 2 in the main text. Figure S5. Weighted UniFrac clustering of Trifolium root and soil samples linked to differences in phyla abundances. Figure S6. Clone library sequences clustering to OTUs from the root community profiles. Figure S7. Quantitative and qualitative comparisons of soil extract inoculum α-diversity to native field soil. Figure S8. Abundant root OTUs in the soil extract microcosm experiment. Figure S9. PCoA plot of inoculum, substrate, and root samples from the soil extract microcosm experiment clustered with native field soil and climate chamber root samples and a comparison between microcosm and climate chamber root communities. Figure S10. Box plot of sequencing depth across climate chamber and natural site root and soil samples. Table S1. ANOVA table of α-diversity analysis. Table S2. Taxonomy, OTU ID, and counts of Trifolium RootOTUs with potential genus function and literature references. Table S3. Temperature and light program used in the climate chamber growth and microcosm experiments. Table S4. PCR cycling conditions used in generating amplicons for MiSeq, isolate analysis, and the clone library. (DOCX 2200 kb) [file 40168_2016_220_MOESM1_ESM.docx]

**ADDITIONAL FILE 1: SUPPLEMENTARY INFORMATION**

Deciphering composition and function of the root microbiome of a legume plant

Kyle Hartman, Marcel GA van der Heidjen, Valexia Roussely-Provent, Jean-Claude Walser, Klaus Schlaeppi

**SUPPLEMENTARY METHODS**

*16S rRNA community profiling*

*DNA extraction:* Upon harvest, the replicate root samples from the climate chamber and natural site growth experiments were flash-frozen in liquid Nitrogen and stored at -20ºC until DNA extraction. The root samples were lyophilized and ground to a powder in 2 mL microcentrifuge tubes with one small tungsten bead and a spoonful of glass sand using a Tissue Lyser II (Qiagen, Hilden, Germany; with 2 cycles of 30 seconds at 30 Hz). Genomic DNA was extracted from 500 mg of root (dryweight) and soil (freshweight) subsamples with the FastDNA® SPIN Kit for Soil (MP Biomedicals, Solon, OH, USA) according to the manufacturer’s instructions. Extracted DNA was quantified using a Quant-iT Picogreen dsDNA Assay Kit (Invitrogen, Eugene, OR USA) on a Varian Cary Eclipse fluorescence spectrometer (Agilent Technologies, Santa Clara, CA USA) and diluted to 10 ng µL^-1^.

*Generation of sequencing amplicons with PCR:* Each 20 µL reaction contained 1x 5PRIME Hot MasterMix (5 PRIME, Gaithersburg, MD USA), 0.3% BSA, 200 µM of each primer (799F, 5’-AACMGGATTAGATACCCKG-3’, [1]; 1193R, 5’-ACGTCATCCCCACCTTCC-3’, [2]) and 30 ng or 10 ng of DNA template for root and soil reactions, respectively. All reactions were performed in an iCycler instrument (BioRad, Hercules, CA, USA) with the cycling conditions given in Table S4. Quadruplicate reactions were pooled, inspected on a 1% agarose gel, and purified using the NucleoSpin PCR purification kit (Machery-Nagel, Düren, Germany). The entire volume of the purified reaction was loaded on a 1% agarose gel, and the ~450 bp amplicon band was cut from the gel. The gel slices were purified using Ultrafree-DA centrifugal filter units (Millipore, Billerica, MA, USA) and quantified using the Picogreen assay described above. The individual samples were pooled in equal amounts into a single 2 mL tube. The volume of the library was reduced with the NuceloSpin PCR purification kit and further purified twice using the Agencourt AMPure XP kit (Beckman Coulter, Brea, CA, USA).

*16S rRNA sequencing:* Preparation of the amplicon library for community profiling was conducted as follows: The TruSeq DNA Sample Prep Kit v2 (Illumina, San Diego, CA, USA) was used following the manufacturer’s instructions. Briefly, the amplicon samples were end-repaired and polyadenylated. TruSeq adapters containing the index for multiplexing were ligated to the amplicon samples. The ligated samples were run on a 2% agarose gel and the desired fragment length were excised (50bp +/- the target fragment length). DNA from the gel was purified with MinElute Gel Extraction Kit (Qiagen, Hilden, Germany). Fragments containing TruSeq adapters on both ends were selectively enriched with PCR using 4 cycles. The quality and quantity of the enriched libraries were validated using Qubit® (1.0) Fluorometer and the Tapestation (Agilent Technologies, Santa Clara, CA USA).  The libraries were normalized to 4nM in Tris-Cl 10 mM, pH8.5 with 0.1% Tween 20. The library was sequenced on the Illumina MiSeq Personal Sequencer (Illumina, San Diego, CA, USA) using a 600 cycle v3 Sequencing kit (Cat n° MS-102-3003), Paired-end 2x 300 bp sequencing mode.

*Normalization of OTU counts with rarefaction*

Because we found significant differences between the mean sequencing depths per sample group (Kruskal-Wallis test, *p*=0.008; Figure S10), we chose to rarefy the OTU table and tested for differentially abundant OTUs using non-parametric Mann-Whitney-U tests. This approach is recommended by Weiss *et al.,* [3] who demonstrated that rarefying erases artifacts due to the different sequencing depths of sample groups better than other normalization techniques. For the detection of differentially abundant OTUs however, rarefied data precludes the use of dedicated statistics such as DESeq2 [4] or metagenomeSEQ [5]. However, non-parametric tests on rarefied data offer robust and specific detection of differentially abundant OTUs [3].

*Statistical analysis of community profiles*

All analyses were performed using R v3.1.2 [6] and the specific R and Biocondutor packages listed below. The OTU and taxonomy tables were filtered to exclude OTUs classified as Eukaryotes, chloroplasts, and mitochondria. The OTU table was rarefied to 20,000 sequences per sample using the R-package *vegan* v 2.3-5 [7]; Figure S10, see method above). We then calculated the relative abundance of each OTU by dividing the number of counts of an OTU in a sample by the total number of counts in that sample, and we expressed the proportions as percentages. All statistical analyses were performed on log_2_+1 transformed relative abundance OTU counts.

*Alpha and beta diversity:* Rarefaction analysis was performed in QIIME v,1.8 [8] on the filtered OTU table (exported from R for this purpose) from 2,000 to 100,000 sequences with a step size of 2,000 and 100 iterations at each sequencing depth. Estimates of alpha diversity (OTU richness and Faith’s Phylogenetic Diversity, PD [9] were calculated using the R-package *picante* v1.6-2 [10]. Differences in alpha diversity measures were tested using Two-way ANOVA with the model *Alpha Diversity Measure ~ Sample Type * Growth Condition.* To quantify the major variance components of beta diversity between root and soil samples, we performed PCoA on weighted UniFrac distances [11] calculated from the phylogenetic tree using PyNAST [12] aligned sequences and FastTree [13] as implemented in QIIME v1.8*.* The beta diversity analysis was performed with the Bioconductor package *phyloseq* v1.14 [14].

*Defining the enriched and abundant members of the root microbiome:* We defined the root enriched OTUs (significantly higher abundance in root compared to soil samples) by utilizing non-parametric Mann-Whitney tests, and we considered the OTUs with a >2-fold change between root and soil samples and *P*<0.05 (FDR corrected) to be the root enriched community. We further defined the abundant members of the Trifolium root microbiome (RootOTUs) as OTUs having a mean relative abundance of ≥0.1% across all samples. This abundance threshold is similar to previous studies [15–18]. All *P*-values were adjusted for multiple comparisons with the FDR correction using the Benjamini-Hochberg method [19]. Four RootOTUs could not be taxonomically assigned using SILVA and were assigned using the 16S ribosomal RNA database with NCBI BLAST. The R code and all necessary input files are available in Additional file 4.

*Bacteria reference stock*

*Root processing for bacteria isolation:* Freshly harvested 5 cm root fragments of 9 plants were cut into smaller segments of 1-3 cm. The cut and mixed root segments were then divided into three sterile 50 mL tubes containing 25 mL sterile phosphate-buffered saline buffer supplemented with 0.05% Tween 20 (PBS-T buffer) and vortexed at maximum speed for 30 sec. The roots were removed from this first washing step and transferred to new 50 mL tubes containing fresh 25 mL PBS-T buffer and shaken at 28°C and 150 rpm for 20 min. After this second washing step, the root segments were transferred to a sterile Petri dish and divided equally between 24 2 mL microcentrifuge tubes each containing 750 µL sterile PBS-T buffer, one large Tungsten bead (⌀ 2 mm) and one small spoonful of glass beads (⌀ 0.8 mm). The samples were lysed with a TissueLyser II (Qiagen, Hilden, Germany) for two cycles of 2.5 min at 30 Hz. We created the root slurry for plating by pooling the contents of all tubes into one meta-sample and filtering it through a sterile 250 µm sieve to remove the lysing beads and large root debris.

*Bacteria isolation:* The root slurry was serially diluted and 20 µL of the 10^-4^ to 10^-7^ dilutions were plated onto Flour medium (FM) agar [20] plates amended with 10 µg mL^-1^ Cycloheximide (to inhibit fungal growth; Sigma Aldrich, St. Louis, MO USA). The plates were incubated at 28˚C for 1-10 days. Single colony forming units were selected and sub-cultured three times on FM plates. The isolates were collected as the bacteria reference stock in 96-well deep-well culture plates containing 1 mL FM medium and were duplicated for PCR-based taxonomy identification (see below) or supplemented with 20 % glycerol (v/v final) for long-term storage at -80°C.

*Isolate identification:* The isolates were grown in liquid FM in 96-well plates until turbid and a 100 µL subsample taken and centrifuged (5min 16 060 x g) to pellet microbial biomass. The supernatant was removed, replaced with sterile H_2_O, and DNA extracted by boiling at 99ºC for 10 min to lyse the cells. The plates were centrifuged (5 min 16,060 x g) to pellet cell debris and the supernatant used as DNA template in PCR reactions. Each 20 µL PCR reaction per isolate contained 1 U Phusion High Fidelity DNA Polymerase, 1x HF buffer, 200µM dNTPs, 30nM of each primer (27F, 5’-AGAGTTTGATCCTGGCTCAG-3’, [21]; 1401R, 5’-CGGTGTGTACAAGGCCC-3’, [22] and 3 µL of template DNA. All reactions were performed in an iCycler instrument (BioRad, Hercules, CA, USA) with the cycling conditions given in Table S4. PCR amplicons were verified on a 1% agarose gel. The reactions were purified and sequenced using the Sanger method with 1401R as the sequencing primer by Microsynth AG (Balgach, Switzerland).

*Quality filtering and taxonomic classification of bacteria isolate sequences:* The resulting AB1 sequencing files (available in Supplementary data 5) were converted into FASTQ file format using EMBOSS v6.6.0 [23], and degenerate nucleotides were re-assigned with Seqtk (https://github.com/lh3/seqtk). The sequences were then re-orientated to the 5’-3’ direction using FASTX v0.0.13 (http://hannonlab.cshl.edu/fastx_toolkit/). Sequences were quality filtered by trimming 50 bp from the 5’ and 3’ ends and then progressively trimming nucleotides from both ends at a mean phred score <25 (window size 5, step size 2). Finally, sequences < 700bp or with a mean phred score < 25 were discarded. Quality filtering was performed using PRINSEQ v0.20.4 [24]. Quality sequences were used for taxonomy assignment using the RDP classifier against the SILVA (v119) [25] database as implemented in QIIME v1.8. 23 isolates could not be assigned using SILVA and were further classified against the 16S ribosomal RNA sequences database using NCBI BLAST.

*Phylogenetic tree*: Bacteria isolate sequences were aligned using the PyNAST algorithm in QIIME v 1.8*.* The phylogenetic tree file was generated using FastTree imported into R, and visualized using the *plot.phylo* function in the R package *ape* [26].

*Mapping isolates to OTUs*

To cross-reference cultivation independent and dependent efforts we mapped the 16S rRNA sequences of the isolates in the Trifolium reference stock to the OTU representative sequences obtained from the community profiling. The quality-filtered, full-length sequences of the isolates were trimmed 5‘ of the 799F primer site using FLEXBAR v2.4 [27] and trimmed to 360 bp to identify the same region of the 16S rRNA operon as used for community profiling. The trimmed isolate sequences were then mapped to the OTU representative sequences at ≥97% sequence similarity using UPARSE [28]

*Estimating within-nodule diversity with a clone library*

*Nodule harvest, DNA extraction, clone library preparation:* We harvested nodules from a separate Trifolium growth experiment conducted and harvested as previously mentioned, except the harvested roots were preserved in 50% EtOH and stored at room temperature until harvesting of the nodules. We aseptically cut 30 nodules from 10 plants and surfaced sterilized them by soaking for 5 min in 5% household bleach and rinsing thoroughly with sterile distilled H_2_O. The 30 nodules were then separated into 3 samples of 10 nodules each. DNA was extracted from each sample with the FastDNA® SPIN Kit for Soil (MP Biomedicals, Solon, OH, USA) according to the manufacturer’s instructions. An 16S rRNA amplicon for cloning was generated using the PCR primers 27F (5’-AGAGTTTGATCCTGGCTCAG-3’; [21] and 1401R (5’-CGGTGTGTACAAGGCCC-3’; [22]). PCR was conducted in 50 µL reactions for each sample and contained 1 U Phusion High Fidelity DNA Polymerase (Thermo Scientific, Waltham, MA, USA), 1x HF buffer, 200 µM dNTPs, 300 nM of each primer and 3 µL of template DNA. All reactions were performed in an iCycler instrument (BioRad, Hercules, CA, USA) with the cycling conditions given in Table S4. PCR amplicons were verified on a 1% agarose gel and the remaining volume purified using the QIAquick PCR Purification Kit (Qiagen, Hilden, Germany). The purified 16S amplicons were cloned in vectors and subsequently chemically transformed in One Shot® Mach1™-T1^R^ Chemically Competent *E. coli* using the Zero Blunt TOPO PCR Cloning Kit (Invitrogen, Eugene, OR USA) according to the manufacturer’s instructions. Vectors from 96 individually picked colonies were isolated and amplicons sequenced using the Sanger method with the gene-specific primer 1401R by Microsynth AG (Balgach, Switzerland). The raw sequencing files are available from the authors upon request.

*Microcosm Experiments*

*Design and construction of microcosms:* We modified Magenta GA-7 boxes (Sigma Aldrich, St. Louis, MO USA) to use as microcosms and filled them with 70 g of a calcined clay The experimental substrate had the following physicochemical characteristics: pH 7.3; 96/0/0 % clay/humus/silt; 0.9/ 4.0/ 44.0 mg/kg N/P/K (measured in 1:10 water extract by Eric Schweizer AG, Thun, Switzerland). The lids of the microcosms were modified to have four holes (⌀ 1.5 cm) to permit air exchange. After autoclaving, they were assembled in a flow bench as follows: three holes were sealed with sterile gas-permeable foil and the fourth one was filled with a microbiological foam stopper (possibility to water the plants with a syringe) (Figure S1a,b).

*Soil extract experiment:* We prepared a soil extract from the same batch of experimental soil that we used for the other experiments of this study. We aimed at a procedure that extracts the microbiota from a soil, removes structural and nutritional properties of the soil and thereby presents a “clean” microbial inoculum for subsequent experiments. We blended for each a subsample (5 g) of the experimental soil in 10 ml sterile PBS-T buffer in a laboratory blender (Polytron, Kinematica, Lucerne, Switzerland; setting 3 for 30 seconds) and collected the supernatant after centrifugation (2 min 3,220x g). This extraction step was repeated three times and the supernatants combined. The blender was sterilized by soaking in 5% bleach for 5 min, 70% ethanol for 5 min, followed by two rinses in sterile H_2_O. We inoculated 200 µL of the soil slurry to 50 mL of 15% Hoagland solution [29] amended with 10 µg mL^-1^ Cycloheximide to reduce fungal growth and incubated for 72 hours in a 28˚C incubator at 150 rpm. We chose this approach to reduce fungal growth, enrich the soil extract for bacteria and because we wanted to expose the soil microbiota to the nutrient conditions of the future microcosm experiments. After this preconditioning step, we centrifuged the liquid cultures (5 min, 3 220x g) to pellet microbial biomass, discarded the supernatant, and then re-suspended the microbiota in 50 mL of fresh 15% Hoagland solution. We estimated the bacterial cell number with plating serial dilutions on FM medium to 1.4*10^9^ cells mL^-1^. The fresh soil microbiota extract was maintained at 4ºC until inoculation to the microcosm. Aliquots of the soil microbiota extract were sampled for community analysis to quantify the inoculum at the start of the experiment.

In a sterile flow bench, we inoculated 70 mL of 15% Hoagland solution containing 1*10^6^ cells mL^-1^ of soil microbiota to the substrate in the microcosms, planted four Trifolium seedlings in each microcosm, and closed them with the modified lids (Figure S1c). The microcosms were maintained in a climate chamber at constant, light, temperature, and humidity conditions (Table S3), and after 25 days, we collected root (5cm) and substrate samples (Figure S1d). Finally, we determined the community profiles of soil extract samples (4 independent extractions as described above), root samples, and substrate samples using the approach described above.

*Simplified bacteria community experiment:* We used four bacteria strains from the reference stock isolate collection to conduct a microcosm experiment to assess their effects on plant growth when inoculated individually or in combination. We performed three replicate experiments, each having 24 microcosms (6 treatments * 4 replicates). In a sterile flow bench, we inoculated the microcosms of each bacteria treatment with 70 mL of 15% Hoagland solution containing 1*10^6^ cells mL^-1^ (OD_600_). Replicates of the combination treatment were inoculated with 70 mL of 15% Hoagland’s solution containing equal amounts of all four bacteria strains to reach a final concentration of 1*10^6^ cells mL^-1^. 70 mL of nutrient solution without bacteria served as the control. We planted four Trifolium seedlings in each microcosm, and closed them. The microcosms were maintained in a climate chamber at constant, light, temperature, and humidity conditions (Table S3). After 25 days, we collected root (5cm) samples from each replicate microcosm and quantified bacteria colonization on the roots.

*Quantification of root bacteria colonization:* At the harvest of each simplified community experiment, we determined the level of bacterial colonization in the root. One root from each replicate was cut from the plant and homogenized in 1.5 mL Eppendorf tubes containing 1mL 10mM MgCl_2,_ glass beads (⌀ 0.8mm), and one tungsten bead (⌀ 2mm) with the Tissue Lyser II instrument for 3 minutes at 15Hz. The resulting root slurry was serially diluted from 10^-1^ to 10^-6^ and 10µL of each dilution plated on FM [20] agar plates amended with 10µg mL^-1^ Cycloheximide. The plates were incubated for 24 hours at 28°C before visual identification and counting of individual colonies to determine cell density of the inoculated bacteria.

*Statistical analysis of simplified community experiments* We assessed the effect of the bacteria treatments in the three replicate experiments using Two-way ANOVA with the following model: *Shoot Fresh Weight ~ Bacteria treatment * Experiment.* Values for shoot fresh weight were log transformed to the assumptions for ANOVA. Significant differences between the different treatments were assessed with Tukey’s HSD test and were considered significant at *P* < 0.05. We subsequently determined the proportion of sequences from the tested strains in the experimental inoculum and on the root samples by applying a relative abundance threshold of ≥0.1% across the inoculum and root samples separately.

**SUPPLEMENTARY RESULTS**

*Exploring nodule diversity with a clone library*

The high abundance of *OTU1* in the overall root community suggested that these sequences originated from rhizobia bacteria present in root nodules. We tested this hypothesis by Sanger sequencing of a 16S rDNA clone library prepared from separately collected and surface-sterilized nodules, and we mapped the nodule-derived sequences to the reference OTUs of the community sequencing. The 95 nodule-derived sequences comprised 10 unique sequences that clustered into three OTUs (Figure S6). The majority of sequences (#91, 6 unique sequences) indeed clustered to *OTU1*, and 3 sequences (all unique) clustered to *OTU3*, a member of the γ-Proteobacteria. A single unique sequence with a 98% match to *Rhizobium leguminosarum* bv. viciae (NCBI BLAST) could not be clustered to a root or soil OTU in our dataset. Besides confirming that *OTU1* represents the rhizobia bacteria in the nodules, we noted some within-nodule diversity at ≥97% sequence similarity (Figure S6).

*Microcosms for plant-microbiota experiments*

We developed a microcosm system where we grew Trifolium plants in polycarbonate boxes that were supplemented with a calcined clay-based growth substrate and a mineral nutrient solution (Figure S1a-d). We tested the suitability of these microcosms as an experimental system to investigate plant-microbiota interactions. We inoculated soil extract to the microcosms, planted Trifolium, and measured the community assembly on the roots after 25 days of incubation (see Supplementary methods for details). Of note, the microcosms were maintained in the same climate chamber, under the same conditions, and the soil extract was prepared from the same soil batch that we used for the climate chamber experiments with soil-grown Trifolium. Goals of this experiment were to evaluate soil extract as start inoculum compared to native field soil and to define the Trifolium root microbiome in the microcosms. We collected root, substrate, and soil extract samples and determined the bacterial communities using the same approach as described above. The microcosm experiment yielded 156,850 high-quality, non-chimeric sequences with a median of 10,062 (range 1,251 – 20,466) sequences per sample (Additional file 2). For community comparison we sub-sampled the data to 3,000 sequences per sample, removing one substrate sample (only 1,251 sequences) from the analysis.

Initially, we prepared four replicate soil extracts and evaluated them as start inoculum by comparing its community composition to that of the native soil from which it was prepared. To this end, we quantified bacteria OTU richness and found 596 ± 2 OTUs (mean ± s.e.m) in the experimental soil samples and 544 ± 12 OTUs in the soil-derived extract samples. OTU richness only differed slightly between native soil and soil extract samples (Welch’s t-test; t=4, *p*=0.02 Figure S7a) revealing that our soil extract procedure recapitulated a large portion of the bacterial richness of the natural soil. While native soil and soil extract communities were qualitatively similar, we noted quantitative differences in the taxonomic composition of soil and soil extract communities (Figure S7b). Thus, soil extract is a reproducible start community with bacteria richness comparable to natural field soil, and these results suggest that it presented a suitable tool to inoculate the microcosms.

Subsequently, we analyzed the bacterial communities in the microcosms that formed the soil extract inoculum. We were interested how the root communities in the microcosms compare to those of natural soil-grown Trifolium and whether a selection of bacteria from the substrate of the microcosm to plant roots occurs. We compared the bacterial communities of microcosms (soil extract, substrate, and root samples) and climate chamber (soil and root samples) experiments using PCoA of weighted UniFrac distances to separate the driving factors explaining community differences. There was a distinct separation along PCo axis 1 (explaining 66.6% of the overall variation) between the microcosm and natural soil-grown root samples (Figure S9a). Because the roots in the microcosms were exposed to basically the same microbiota as the roots grown in native soil, this indicates that the composition of the root communities primarily responds to the different physicochemical properties of the growth environment. PCo axis 2 explained 16% of the overall variation and separated the soil and soil extract samples from the root and the microcosm substrate samples. We interpreted the clustering of soil extract and natural soil samples as further support that the soil extract preparation procedure resulted in a “soil-like” start community for the microcosm experiments. This experiment also provided insights into community dynamics when a soil microbiota is introduced into the microcosms: the soil extract inoculum and the microcosm substrate samples clustered distantly and clearly apart from each other in the ordination space revealing that the introduced soil microbiota underwent a substantial community rearrangement in the new environment. Additionally, we noted a subtle separation between clusters of substrate and root samples of the microcosm experiment, suggesting that, like plants cultivated in natural soil, a selection for a root-specific community also occurs in our microcosm system.

In the microcosms, observed richness of the Trifolium root microbiome was 121 ± 11 OTUs (mean + s.e.m). We identified 34 OTUs whose mean relative abundance across all samples was ≥ 0.1%, and these accounted for 95.9% of rarefied microcosm root sequences (Figure S8). The substantial differences between root communities in microcosms and in natural soil appear to be at least partly the result of a differential recruitment of rhizobia as evidenced by a lower abundance of *OTU1* in microcosm roots compared to the root communities in natural soil (Figure S9b). The soil extract experiment was also intended to identify which OTUs successfully establish under the conditions in the microcosms, which in turn could serve as a rational to choose strains of the reference stock for inoculations to the microcosms (Figure S8).

In summary, the microcosm experiment indicated that soil extract was qualitatively similar to that of normal field soil and served as a diverse start community for microcosm experiments. The inoculated bacteria community underwent substantial community changes in the microcosms, reflected by the distinct clustering of the different sample types. Natural soil and microcosm root communities were qualitatively and quantitatively dissimilar, highlighting the strong community-deterministic effects of the physiochemical characteristics of the growth substrate.

**SUPPLEMENTARY DISCUSSION**

*Root microbiome assembly in microcosms*

We produced the soil microbiota with a custom extraction protocol, which was designed to separate the microbes from the physical and chemical components (e.g. nutrients) of the soil we extracted. First, we evaluated whether the soil extraction protocol yielded a microbial inoculum similar in diversity compared to the native soil from which it was extracted. We found with regard to α-diversity that the soil extract was only slightly different in richness from that of the native field soil (Figure S7a). We interpreted this qualitative similarity that our extraction method provides a rich and diverse start community to inoculate microcosms. We found, however, substantial shifts in the taxonomic composition in the soil extract inoculum compared to the natural soil (Figure S7b). We think that the homogenization of the soil as well as the conditioning step (adapting the soil microbes to the plant nutrient solution and incubation with fungicide to counter-select fungi), represent physical and chemical disturbances that disrupt the equilibrium between members of the native soil microbiota and therefore, influence the relative proportions in the inoculum. We assumed that the inoculation of the soil extract to the microcosms subjects the soil microbiota to an ectopic environment (clay substrate and nutrient solution), and we speculated that the community would find a new equilibrium (different community composition) adapted to the new physicochemical conditions. Indeed, we found a distinct clustering between soil extract and substrate samples (Figure S9a), evidencing that the inoculated soil microbiota underwent a substantial community rearrangement in response to the new conditions in the ectopic environment. Also because of this observation, we conclude that for a start inoculum the presence/absence of taxa is more important than their relative abundances.

The main interest of the soil extract experiment was to follow the assembly of root microbiome in the microcosms and to compare its composition to the one of roots in native soil. Despite that the roots in microcosms and in natural soil were largely exposed to the same soil microbiota and grown under the same controlled climatic conditions, we found that their bacteria communities clustered distantly and clearly apart (Figure S9a). A first explanation is that the strong compositional differences reflect the response of the microbes to the new physicochemical conditions in the microcosms. This explanation conceptually reminds the recurrent observation that the type of soil in which plants root primarily drives the composition of root bacteria communities [16,17,30–32]. In soil, the biogeography of the microbes is mainly determined by edaphic factors and complex interactions between microorganisms [33,34]. In the microcosms, even with the addition of the nutrient solution, the environmental conditions were limited in carbon and nitrogen compared to the native soil and therefore, these conditions potentially exerted a strong selective pressure on the inoculated community and favored those taxa that could quickly adapt to new conditions. Following this logic, edaphic differences between the native organic soil and the predominately mineral substrate would explain the distant clustering and compositional differences of the root communities in soil and microcosm samples (Figure S9a). A second explanation refers to possible effects arising from the difference in duration between the microcosm and natural soil experiments. With the natural soil plants rooted nearly 5 weeks longer than the microcosm grown plants, it is possible that we harvested the microcosm grown plants while dynamic processes of root microbiome assembly were still occurring. Edwards *et al.,* [30] demonstrated that axenic rice seedlings once transplanted into soil begin to assemble an endophyte community within 24h, and that after nearly 2 weeks, rhizoplane and endosphere communities are similar to communities of the same compartment in plants that have been growing for longer. This finding suggests that the bacteria community in microcosm roots would have reached a reasonably representative stage at the time of harvest. However, we observed that root colonization by rhizobia was lower in microcosms compared to soil grown plants (Figure S9b), and therefore future time-course studies are needed to determine the timing when Trifolium roots reach full nodulation and a stable equilibrium in microbiome composition.

We noted in our ordination analysis that the microcosm substrate samples clustered slightly apart from the microcosm root samples (Figure S9a). One interpretation is that we observed a root selection effect in the microcosms that is induced by exudates secreted from Trifolium roots. The subtle shifts in community compositions may have resulted from certain taxa that proliferated better in response to root exudates, or a competitive advantage of some microbes to colonize the specialized conditions of the root, or a combination of both [35]. Additionally, we noticed that all microcosm root samples clustered closely with each other indicating that the root microbiome established in a reproducible manner in the microcosm conditions. It seems plausible that the homogenous structural and nutritional conditions in the microcosms result in fewer microbial niches compared to a complex natural soil and consequently, microbiome assembly occurs with less variation and possibly also of lower diversity. Supporting such an interpretation, Tkacz *et al.,* [36] conducted rhizosphere microbiota transfection experiments and found that the rhizosphere bacterial diversity and variation between samples generally decreased when nutrient poor sand was used as growth substrate compared to compost, which is rich in organic nutrients. In summary, our soil extract experiment revealed for an introduced microbiota that it undergoes substantial community rearrangement in microcosms, that it reproducibly assembles to a stable root microbiome and also that root selection probably occurs under these conditions.

**SUPPLEMENTARY REFERENCES**

1. Chelius MK, Triplett EW. The Diversity of Archaea and Bacteria in Association with the Roots of Zea mays L. Microb. Ecol. 2001;41:252–63.

2. Bodenhausen N, Horton MW, Bergelson J. Bacterial communities associated with the leaves and the roots of Arabidopsis thaliana. PLoS One. 2013;8:e56329.

3. Weiss SJ, Xu Z, Amir A, Peddada S, Bittinger K, Gonzalez A, et al. Effects of library size variance, sparsity, and compositionality on the analysis of micrbiome data. PeerJ Prepr. 2015;

4. Love MI, Huber W, Anders S. Moderated estimation of fold change and dispersion for RNA-seq data with DESeq2. Genome Biol. 2014;15:1–21.

5. Paulson JN, Stine OC, Bravo HC, Pop M. Robust methods for differential abundance analysis in marker gene surveys. Nat. Methods. 2013;10:1200–2.

6. R Core Team. R: A Language and Environment for Statistical Computing. Vienna, Austria: R Foundation for Statistical Computing; 2015.

7. Oksanen J, Blanchet FG, Kindt R, Legendre P, Minchin PR, O’Hara RB, et al. vegan: Community Ecology Package. 2015.

8. Caporaso JG, Kuczynski J, Stombaugh J, Bittinger K, Bushman FD, Costello EK, et al. QIIME allows analysis of high-throughput community sequencing data. Nat. Methods. 2010;7:335–6.

9. Faith DP. Conservation evaluation and phylogenetic diversity. Biol. Conserv. 1992;61:1–10.

10. Kembel SW, Cowan PD, Helmus MR, Cornwell WK, Morlon H, Ackerly DD, et al. Picante: R tools for integrating phylogenies and ecology. Bioinformatics. 2010;26:1463–4.

11. Lozupone C, Knight R. UniFrac: a New Phylogenetic Method for Comparing Microbial Communities. Appl. Environ. Microbiol. 2005;71:8228–35.

12. Caporaso JG, Bittinger K, Bushman FD, DeSantis TZ, Andersen GL, Knight R. PyNAST: a flexible tool for aligning sequences to a template alignment. Bioinformatics. 2010;26:266–7.

13. Price MN, Dehal PS, Arkin AP. FastTree: Computing Large Minimum Evolution Trees with Profiles instead of a Distance Matrix. Mol. Biol. Evol. 2009;26:1641–50.

14. McMurdie PJ, Holmes S. Phyloseq: An R Package for Reproducible Interactive Analysis and Graphics of Microbiome Census Data. PLoS One. 2013;8:e61217.

15. Benson AK, Kelly SA, Legge R, Ma F, Low SJ, Kim J, et al. Individuality in gut microbiota composition is a complex polygenic trait shaped by multiple environmental and host genetic factors. Proc. Natl. Acad. Sci. U.S.A. 2010;107:18933–8.

16. Bulgarelli D, Rott M, Schlaeppi K, Ver Loren van Themaat E, Ahmadinejad N, Assenza F, et al. Revealing structure and assembly cues for Arabidopsis root-inhabiting bacterial microbiota. Nature. 2012;488:91–5.

17. Lundberg DS, Lebeis SL, Paredes SH, Yourstone S, Gehring J, Malfatti S, et al. Defining the core Arabidopsis thaliana root microbiome. Nature. 2012;488:86–90.

18. Bai Y, Müller DB, Srinivas G, Garrido-Oter R, Potthoff E, Rott M, et al. Functional overlap of the Arabidopsis leaf and root microbiota. Nature. 2015;528:364–9.

19. Benjamini Y, Hochberg Y. Controlling the False Discovery Rate: A Practical and Powerful Approach to Multiple Testing. J. R. Stat. Soc. Ser. B. 1995;57:289–300.

20. Coombs JT, Franco CMM. Isolation and Identification of Actinobacteria from Surface-Sterilized Wheat Roots. Appl. Environ. Microbiol. 2003;69:5603–8.

21. Lane D. 16S/23S rRNA sequencing. In: E S, Goodfellow M, editors. Nucleic acid Tech. Bact. Syst. New York, NY: John Wiley and Sons; 1991. p. 115–75.

22. Nübel U, Engelen B, Felske A, Snaidr J, Wieshuber A, Amann RI, et al. Sequence heterogeneities of genes encoding 16S rRNAs in Paenibacillus polymyxa detected by temperature gradient gel electrophoresis. J. Bacteriol. 1996;178:5636–43.

23. Rice P, Longden I, Bleasby A. EMBOSS: The European Molecular Biology Open Software Suite. Trends Genet. 2000;16:276–7.

24. Schmieder R, Edwards R. Quality control and preprocessing of metagenomic datasets. Bioinformatics. 2011;27:863–4.

25. Quast C, Pruesse E, Yilmaz P, Gerken J, Schweer T, Yarza P, et al. The SILVA ribosomal RNA gene database project: improved data processing and web-based tools. Nucleic Acids Res. 2013;41:D590–6.

26. Paradis E, Claude J, Strimmer K. APE: Analyses of Phylogenetics and Evolution in R language. Bioinformatics. 2004;20:289–90.

27. Dodt M, Roehr JT, Ahmed R, Dieterich C. FLEXBAR-Flexible Barcode and Adapter Processing for Next-Generation Sequencing Platforms. Biology (Basel). 2012;1:895–905.

28. Edgar RC. UPARSE: highly accurate OTU sequences from microbial amplicon reads. Nat. Methods. 2013;10:996–8.

29. Hoagland DR, Arnon DI. The water-culture method for growing plants without soil. Circ. Calif. Agric. Exp. Stn. 1950;347:1–32.

30. Edwards J, Johnson C, Santos-Medellín C, Lurie E, Podishetty NK, Bhatnagar S, et al. Structure, variation, and assembly of the root-associated microbiomes of rice. Proc. Natl. Acad. Sci. U.S.A. 2015;112:E911–20.

31. Yeoh YK, Paungfoo-Lonhienne C, Dennis PG, Robinson N, Ragan MA, Schmidt S, et al. The core root microbiome of sugarcanes cultivated under varying nitrogen fertiliser application. Environ. Microbiol. 2015;18:1338–51.

32. Peiffer JA, Spor A, Koren O, Jin Z, Tringe SG, Dangl JL, et al. Diversity and heritability of the maize rhizosphere microbiome under field conditions. Proc. Natl. Acad. Sci. U.S.A. 2013;110:6548–53.

33. Fierer N, Jackson RB. The diversity and biogeography of soil bacterial communities. Proc. Natl. Acad. Sci. U.S.A. 2006;103:626–31.

34. Nannipieri P, Ascher J, Ceccherini MT, Landi L, Pietramellara G, Renella G. Microbial diversity and soil functions. Eur. J. Soil Sci. 2003;54:655–70.

35. Gaiero JR, McCall C a, Thompson K a, Day NJ, Best AS, Dunfield KE. Inside the root microbiome: bacterial root endophytes and plant growth promotion. Am. J. Bot. 2013;100:1738–50.

36. Tkacz A, Cheema J, Chandra G, Grant A, Poole PS. Stability and succession of the rhizosphere microbiota depends upon plant type and soil composition. ISME J. 2015;9:2349–59.

37. Masson-Boivin C, Giraud E, Perret X, Batut J. Establishing nitrogen-fixing symbiosis with legumes: how many rhizobium recipes? Trends Microbiol. 2009;17:458–66.

38. Hahm MS, Sumayo M, Hwang YJ, Jeon SA, Park SJ, Lee JY, et al. Biological control and plant growth promoting capacity of rhizobacteria on pepper under greenhouse and field conditions. J. Microbiol. 2012;50:380–5.

39. Innerebner G, Knief C, Vorholt JA. Protection of Arabidopsis thaliana against leaf-pathogenic Pseudomonas syringae by Sphingomonas strains in a controlled model system. Appl. Environ. Microbiol. 2011;77:3202–10.

40. Adhikari T, Joseph C, Yang G, Phillips D, Nelson L. Evaluation of bacteria isolated from rice for plant growth promotion and biological control of seedling disease of rice. Can. J. Microbiol. 2001;47:916–24.

41. Benítez MS, McSpadden Gardener BB. Linking sequence to function in soil bacteria: Sequence-directed isolation of novel bacteria contributing to soilborne plant disease suppression. Appl. Environ. Microbiol. 2009;75:915–24.

42. Berg G, Opelt K, Zachow C, Lottmann J, Götz M, Costa R, et al. The rhizosphere effect on bacteria antagonistic towards the pathogenic fungus Verticillium differs depending on plant species and site. FEMS Microbiol. Ecol. 2006;56:250–61.

43. Enya J, Shinohara H, Yoshida S, Tsukiboshi T, Negishi H, Suyama K, et al. Culturable leaf-associated bacteria on tomato plants and their potential as biological control agents. Microb. Ecol. 2007;53:524–36.

44. Loiret FG, Ortega E, Kleiner D, Ortega-Rodés P, Rodés R, Dong Z. A putative new endophytic nitrogen-fixing bacterium Pantoea sp. from sugarcane. J. Appl. Microbiol. 2004;97:504–11.

45. Oliveira CA, Alves VMC, Marriel IE, Gomes EA, Scotti MR, Carneiro NP, et al. Phosphate solubilizing microorganisms isolated from rhizosphere of maize cultivated in an oxisol of the Brazilian Cerrado Biome. Soil Biol. Biochem. 2009;41:1782–7.

**SUPPLEMENTARY FIGURES**


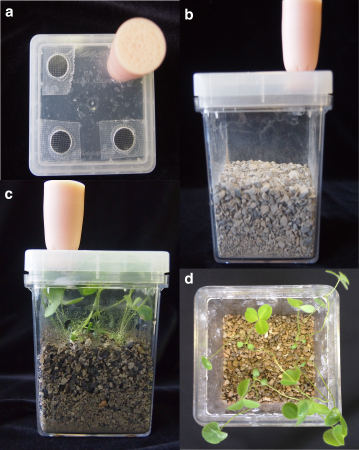


**Figure S1: A microcosm system for testing plant-microbe interactions.** **(a)** Three of the four holes in the lid are covered with a sterile, gas-permeable foil to allow air exchange. The fourth hold is plugged with an autoclaved foam stopper to allow watering of the boxes with a syringe and needle during the experiment. **(b)** The microcosms are filled with calcined clay as a growth substrate. **(c)** The microcosms can support 4 plants during the 25-day experimental period. **(d)** Top down view of 25-day old Trifolium prior to harvest.


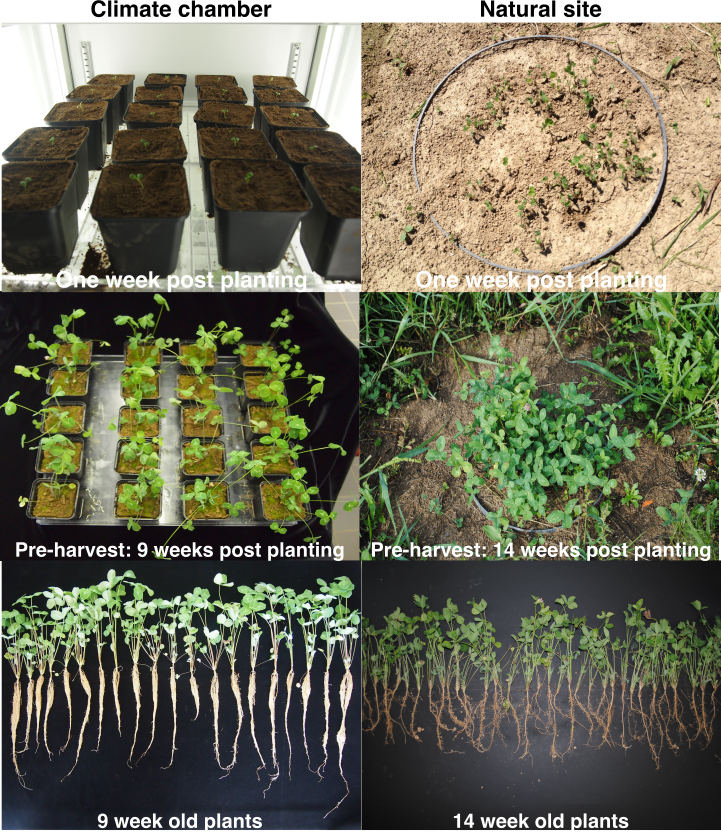


**Figure S2: Cultivating Trifolium for root microbiome profiling and reference stock isolation.** Trifolium cultivated under controlled conditions in individual pots filled with the experimental soil in the climate chamber after one week (top) and 9 weeks of growth (middle). Trifolium cultivated in the experimental soil under natural conditions after one week (top) and 14 weeks of growth (middle). Plants were harvested when they reached a similar phenotypic stage (bottom photos).

**Figure S3: Effects of sample type and growth condition on within sample diversity, as measured by OTU richness, Faith’s Phylogenetic Diversity and Shannon Evenness.** The rarefaction curves in the first row are means of 100 iterations from 2,000 to 100,000 sequences per soil sample (solid lines) and root samples (dashed lines) in the climate chamber (green) and natural site (blue). The red dashed line indicates the rarefaction depth of 20,000 sequences per sample applied to the dataset. The boxplots in the second row show the alpha diversity measures for the rarefied dataset at 20,000 sequences per sample. The climate chamber (green) and natural site (blue) growth conditions are indicated within each sample type. The results of the Two-way ANOVA of the effects of sample type and growth condition are show in Table S1.

**Figure S4: Experimental variation in climate chamber root samples**. Same unconstrained PCoA ordination as depicted in Figure 2 in the main text colored by individual growth experiment in the climate chamber (CC1-CC5) or experimental plot in the natural site (NS1-NS3).

**Figure S5: Differences in β-diversity linked to differences in taxonomy.** The dendrogram of weighted UniFrac distances demonstrates the distance between root and soil samples. The stacked barplots show the relative abundances of the 15 most abundant phyla in the soil samples (solid brown line) and the root samples (dashed green line) of both natural site (NS) and climate chamber (CC) growing conditions.

**Figure S6: *OTU1* inhabits root nodules of *Trifolium*.** The outer ring represents the number of sequences from the clone library clustering to an OTU from the community profile. The inner ring represents the number of unique sequences within the respective OTU.

**Figure S7: The soil extract captures the diversity of the experimental soil. (a)** Means + s.e.m of observed OTU richness of experimental soil samples (n=3) and soil extract inoculum from the microcosm experiments (n=4). **(b)** Heatmap comparing the relative abundances of the shared phyla between the unplanted experimental soil and soil extract samples.

**Figure S8:** **Abundant bacteria community of microcosm roots.** Relative abundances of abundant bacteria OTUs (RA > 0.1%) associated with roots of microcosm grown Trifolium inoculated with a diverse bacteria community from the soil extract. Blue bars indicate OTUs for which an isolate is present in the reference stock, with the number of isolates available for each OTU indicated below each bar.

**Figure S9: Conditions in the microcosms create unique communities. (a)** Unconstrained PCoA plot on weighted UniFrac distances of microcosm inoculum (using soil extracts as inoculum), substrate, and root samples (MB Root), the experimental soil, and the climate chamber root samples (CC Root). **(b)** Relative abundances for OTUs having a mean overall abundance of at least 0.1% across all samples. The soil extract (SEROOT) and climate chamber (CCROOT) sample cluster dendrogram is based upon weighted UniFrac distances. OTUs are ordered according to their relative abundance in climate chamber sample

**Figure S10: Sequencing depth varied significantly among sample types.** Distribution of 16S amplicon sequence counts for climate chamber (CC) and natural site (NS) soil and root samples. Significant differences in sequencing depth required rarefaction of the dataset to 20,000 sequences per sample. See Supplementary Methods for more information.

**SUPPLEMENTARY TABLES**

**Table S1:** Two-way ANOVA analysis of alpha diversity. ANOVA table showing the effects of sample type (root or soil), growth condition (climate chamber or natural site) and their interaction on alpha diversity in the rarefied community as measured by OTU richness, Faith’s Phylogenetic Diversity, and Shannon Evenness. Data are presented in Figure S4.

|  | **OTU Richness** | | | **Faith’s Phylogenetic Diversity** | | | **Shannon Evenness** | | |  |
| --- | --- | --- | --- | --- | --- | --- | --- | --- | --- | --- |
| ***Factor*** | **df** | **F** | ***p*** | **df** | **F** | ***p*** | **df** | **F** | ***p*** | |
| Sample Type | 1, 35 | 450.43 | ***<0.001*** | 1, 35 | 361.71 | ***<0.001*** | 1, 35 | 175.25 | ***<0.001*** | |
| Growth Condition | 1, 35 | 4.71 | ***0.04*** | 1, 35 | 3.25 | 0.08 | 1, 35 | 3.29 | 0.08 | |
| Sample Type * Growth Condition | 1, 35 | 2.90 | 0.10 | 1, 35 | 1.17 | 0.29 | 1, 35 | 1.35 | 0.25 | |

**Table S2:** Taxonomic assignments of the 15 RootOTUs, the abundant (>0.1% RA) and enriched OTUs of the *Trifolium* root microbiome. OTU IDs in bold indicate a culturable member with at least one isolate present in the isolate collection.

| **Phylum** | **Class** | **Order** | **Family** | **Genus** | **OTU ID** | **Reported benefit to host plant** | **Reference** |
| --- | --- | --- | --- | --- | --- | --- | --- |
| ***Proteobacteria*** | *α-* | *Caulobacterales* | *Caulobacteraceae* | *Caulobacter* | *OTU44* | - | - |
|  |  | *Rhodospirillales* | *Rhodospirillaceae* | *Magnetospirillum* | *OTU37** | - | - |
|  |  | *Rhizobiales* | *Rhizobiaceae* | *Rhizobium* | ***OTU1*** |  |  |
|  |  |  |  |  | *OTU13* |  |  |
|  |  |  |  |  | *OTU10* | Nitrogen fixation | [37] |
|  |  |  |  |  | *OTU545** |  |  |
|  |  |  |  |  | *OTU72* |  |  |
|  |  |  |  |  | *OTU2335* |  |  |
|  |  | *Sphingomonadales* | *Sphingomonadaceae* | *Novosphingobium* | *OTU52* | Bacterial disease suppression | [38] |
|  |  |  |  | *Sphingomonas* | *OTU47* | Bacterial disease suppression | [39] |
|  |  |  |  |  | *OTU93** | Plant growth promotion | [40] |
|  | *β-* | *Burkholderiales* | *Comamonadaceae* | *Pelomonas* | *OTU2* | Fungal pathogen antagonism | [41] |
|  | *γ-* | *Enterobacteriales* | *Enterobacteriaceae* | *Pantoea* | *OTU286*  ***OTU48*** | Fungal pathogen antagonism | [42,43] |
|  |  |  |  |  |  | Nitrogen fixation  Inorganic P solubilization | [44]  [45] |
| ***Firmicutes*** | *Clostridia* | *Clostridiales* | *Syntrophomonadaceae* | *Syntrophomonas* | *OTU28** | - | - |
|  |  |  |  |  | **Total: 15** |  |  |

* Taxonomy assignment with NCBI BLAST

**Table S3:** Growth program for the climate chamber growth experiments. The relative humidity was maintained at 60% during all experiments.

| **Time** | **Temp (ºC)** | **Light Intensity** |
| --- | --- | --- |
| 0600 | 16 | Light 1 |
| 0615 | 16 | Light 2 |
| 0630 | 16 | Light 3 |
| 0645 | 16 | Light 4 |
| 0700 | 25 | Light 5 |
| 2100 | 20 | Light 4 |
| 2115 | 20 | Light 3 |
| 2130 | 20 | Light 2 |
| 2145 | 20 | Light 1 |
| 2200 | 16 | Light 0 |

**Table S4:** PCR cycling conditions. Cycling parameters for the generation of the amplicons for MiSeq sequencing for the community profiling, Sanger sequencing for the identification of the reference stock isolates, and the Trifolium root-nodule clone library.

| **MiSeq Amplicons** | | | | **Isolate Identification** | | | | **Clone Library** | | | |  |
| --- | --- | --- | --- | --- | --- | --- | --- | --- | --- | --- | --- | --- |
| Step | Temperature | Time | Cycles | Step | Temperature | Time | Cycles | Step | Temperature | Time | Cycles | |
| 1 | 98ºC | 30sec | 1x | 1 | 94ºC | 2min | 1x | 1 | 98ºC | 30sec | 1x | |
| 2 | 98ºC | 10sec |  | 2 | 94ºC | 30sec |  | 2 | 98ºC | 10sec |  | |
| 3 | 54ºC | 15sec | 30x | **3** | 52ºC | 30sec | 30x | 3 | 52ºC | 15sec | 30x | |
| 4 | 72ºC | 45sec |  | 4 | 65ºC | 30sec |  | 4 | 72ºC | 45sec |  | |
| 5 | 72ºC | 10min | 1x | 5 | 65ºC | 10min | 1x | 5 | 72ºC | 10min | 1x | |
| 6 | 15ºC | hold |  | 6 | 15ºC | hold |  | 6 | 15ºC | hold |  | |
